# Avidin is evolutionarily conserved in fish but dispensable for development and resistance against *Streptococcus agalactiae* in zebrafish

**DOI:** 10.1002/2211-5463.70320

**Published:** 2026-07-31

**Authors:** Anni K. Saralahti, Markus J. T. Ojanen, Mataleena Parikka, Otto Kauko, Mika Rämet, Vesa P. Hytönen

**Affiliations:** ^1^ Faculty of Medicine and Health Technology Tampere University Finland; ^2^ Turku Bioscience Centre University of Turku and Åbo Akademi University Finland; ^3^ Fimlab Laboratories Tampere Finland

**Keywords:** avidin, bacterial infection, CRISPR/Cas9, development, phylogeny, zebrafish

## Abstract

Biotin is a vital cofactor in cellular carboxylation reactions and avidins are biotin‐binding proteins that are produced by bacteria and fungi but also by egg‐laying vertebrates, including avian species and zebrafish (*Danio rerio*). Although avidins have proposed roles in embryonic development and immunity, their biological significance is poorly characterized. Moreover, besides zebrafish, the presence of avidins in other fish species has not been explored. Here, we cataloged avidins in fish species and demonstrate that avidins exist in lobe‐finned fish (Sarcopterygii), cartilaginous fish (Chondrichthyes) and ray‐finned fish (Actinopterygii), and that the biotin‐binding amino acids in fish avidins are evolutionarily conserved. To evaluate the significance of avidin in embryonic development, we created *avd* knockout (KO) zebrafish and show that zebavidin (zebrafish avidin) is dispensable for fish development. We also assessed the survival of the *avd* KO embryos from *Streptococcus agalactiae* infection and demonstrate that the resistance of the embryos is not altered in the absence of zebavidin. Collectively, we show that avidins exist widely in fish species and provide new insights into avidin biology by demonstrating that zebavidin is not essential for zebrafish development or response to *S. agalactiae*.

## Graphical abstract

The presence of biotin‐binding avidin proteins in fish and their biological significance are poorly characterized. We cataloged fish avidins and demonstrate that they are widely present and evolutionarily conserved. We created *avd* knockout zebrafish and show that zebavidin is dispensable for development and that resistance of *avd* knockout embryos in *Streptococcus agalactiae* infection is not altered. Figure created in biorender: https://BioRender.com/wqfxyc3.

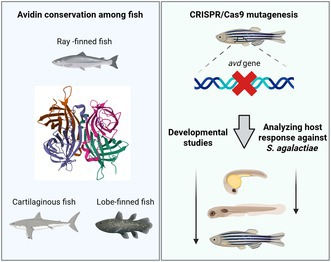

**Abbreviations**
BLAST: Basic Local Alignment Search Tool
cfu: colony forming unit
CRISPR: clustered regularly interspaced palindromic repeats
DIA: data independent acquisition
dpf: days post fertilization
EU: the European Union
gRNA: guide RNA
H&E: hematoxylin and eosin
hpf: hours post fertilization
HPLC: high‐performance liquid chromatography
KO: knockout
LC–MS: liquid chromatography‐coupled mass spectrometry
MEGA: molecular evolutionary genetics analysis
WT: wild‐type
ZFIN: zebrafish information network

Biotin, also known as vitamin H, is an important cofactor that is involved in carbon dioxide metabolism in both multicellular organisms and in microbes [1]. Avidin is a biotin‐binding protein that was initially discovered in the chicken egg white [2], and later shown to be produced in the female reproductive tract of egg‐laying vertebrates [3]. A physiological link between biotin and avidin was established when it was demonstrated that both low biotin and high avidin levels in the egg white cause decreased hatchability in turkeys (*Meleagris gallopavo domesticus*) [4] and that either low levels of dietary biotin or excess egg white/avidin supplementation can have pathological and teratogenic effects in vertebrates, including fish and mice [5, 6, 7, 8, 9]. Despite the established interplay between biotin and avidin, the biological functions of avidins remain poorly understood.

Members of the avidin protein family have been identified in a variety of vertebrates, including amphibians [3, 10, 11] and avian species [10], as well as in fungi [12] and in bacteria [13, 14]. Although the presence of avidins in fish has not been systematically studied, an ortholog for the avian *AVD* gene has been identified in zebrafish (*Danio rerio*) [15], and we have previously reported that it encodes an avidin‐like protein termed zebavidin [16]. More specifically, we demonstrated that zebavidin has highly conserved amino acid residues important for biotin‐binding and that it expectedly binds biotin with high affinity (*K*_D_ = 3.7 × 10^−9^ m). Importantly, *avd* mRNA was enriched in the zebrafish gonads and in the ovum, which suggests functional analogy for zebavidin to its avian homologs.

While the production of avidin in the oviducts of birds can be induced by sex hormones, including progesterone and estrogen [17, 18, 19], inflammation‐inducing conditions, such as tissue injury and bacterial infections, have been shown to increase the production of the protein also in other tissues [20, 21, 22, 23]. Consequently, it has been suggested that avidin functions as a host defense protein by limiting the bioavailability of biotin from pathogenic or competing micro‐organisms [20]. In addition, direct antibacterial properties have been attested for avidin as well as for the prototype bacterial analog, streptavidin, from *Streptomyces avidinii* [13].

Zebrafish is a small teleost that is widely used as a model organism in developmental biology, toxicology and in studying infectious diseases [24, 25, 26]. The benefits of zebrafish as a model organism include its optical transparency during embryogenesis, a fully sequenced and assembled genome and the practicality of studying zebrafish using reverse genetic tools, such as morpholino‐oligonucleotide silencing and clustered regularly interspaced palindromic repeats (CRISPR)/Cas9 mutagenesis [25, 27, 28, 29, 30]. In fact, we have previously used morpholinos to study the impact of *avd* knockdown during zebrafish embryogenesis [16], and have shown that the CRISPR/Cas9 system can be successfully used to understand the impact of host genes on the immune response against streptococcal and mycobacterial infections [31, 32, 33]. However, there are no knockout (KO) animal models for evaluating the biological function of avidins, and the significance of zebavidin during a microbial challenge has not been determined.

Here, we used the Basic Local Alignment Search Tool (BLAST) to identify *avd* genes in fish species and performed phylogenetic analyses to study the evolutionary relationships of fish avidins. To evaluate the importance of avidin for fish development and embryonic survival, we used CRISPR/Cas9 mutagenesis to make an *avd* KO zebrafish line and analyzed whether the absence of zebavidin affects fish development or resistance against *Streptococcus agalactiae*.

## Materials and methods

### Identification of fish avidins

Uniprot‐derived amino acid sequences of chicken (*Gallus gallus*, P02701), *Xenopus tropicalis* (A7YYL1), *Streptomyces avidinii* (P22629), and zebrafish (E7F650) avidin were trimmed to exclude SignalP 6.0‐predicted signal sequences [34] and used to search for avidin‐like proteins and avidin‐encoding genes from jawless (Agnatha, taxid: 1476529), bony (Actinopterygii, taxid: 7898 and Sarcopterygii, taxid: 8287), and cartilaginous fish (Chondrichthyes, taxid: 7777) using the Basic Local Alignment Search Tools (BLAST) BLASTp and tBLASTn [35]. Tetrapoda (taxid: 32523) was excluded from the BLAST searches (a clade within Sarcopterygii). The maximum amount of target sequences was limited to 5000, and BLOSUM62 was used as the scoring matrix. The parameters were adjusted for a short input sequence with an E‐value cutoff of 0.01. Duplicate hits were excluded by searching for overlapping hit IDs with InteractiVenn [36].

### Sequence alignment and phylogenetic analysis

Molecular Evolutionary Genetics Analysis (MEGA, v.11.0.13) was used to analyze the phylogenetic relationships of the BLAST‐derived avidin protein sequences. Here, all BLASTp and tBLAST‐derived sequences were first aligned using the integrated muscle tool with the neighbor joining method for clustering, which was followed by the construction of the phylogenetic tree using the Neighbor joining method and the Bootstrap test. The construction of the phylogenetic tree was repeated twice using a similar methodology, and the hit‐containing FASTA file curated between analyses by removing hits/sequences with < 0.1 similarity. After a second curation, query avidins were included to provide reference points to the phylogenetic tree, and the final phylogenetic tree was constructed using the Maximum Likelihood method. For the alignment and comparison of the specific avidin sequences, the signal sequences were removed, and the alignment was performed using muscle.

### Zebrafish maintenance and ethics statement

The zebrafish maintenance and the experiments were in accordance with the Finnish Act on the Protection of Animals Used for Scientific or Educational Purposes (497/2013) as well as the European Union (EU) Directive on the Protection of Animals Used for Scientific Purposes (2010/63/EU). Experiments were approved by the Animal Experiment Board of Finland (permit for zebrafish maintenance: ESAVI/10079/04.10.06/2015; permit for the experiment: ESAVI/7251/2021). The zebrafish embryos and larvae (also called embryos from this point onwards) were maintained according to standard protocols in E3‐water (5 mm NaCl, 0.17 mm KCl, 0.33 mm CaCl_2_, 0.33 mm MgSO_4_, and 0.0003 g·L^−1^ methylene blue) at 28.5 °C until 5 days post fertilization (dpf). Adult zebrafish were kept in a flow through system (Aquatic Habitats, Florida, USA) with an automated light/dark cycle of 14 h/10 h and fed once a day with Gemma Micro 500 (Skretting, Stavanger, Norway).

### CRISPR/Cas9 mutagenesis

Nonsense mutation carrying *avd* ^tpu12^ mutants were created using previously established protocols [31, 32]. Briefly, guide RNA (gRNA) target sites (1. GAAATAGCTCCCACAATGTCACGG, 2. GCCAAGATGAGTTCTTTAATATGG, 3. GAGTTCTTTAATATGGCATTTGG, 4. GCTCATTGCGCCAAACACCGG, 5. GTGAAGGCGGAAGGCTCGGAGG and 6. GAGGTGTTTACCAGACCGCGG) were identified on the first and second exons of zebrafish *avd* (ENSDARG00000087961) using CHOPCHOP v2 (https://chopchop.cbu.uib.no/) [37], and the suitable gRNAs produced using the MEGAshortscript T7 Transcription Kit (Ambion Life Technologies, CA, USA). Subsequently, wild‐type (WT) AB zebrafish oocytes were injected with 150 pg of gRNA and 300 pg of in‐house produced Cas9‐protein (Protein Services, Tampere University) [32] and the mutagenesis efficiency estimated using the heteroduplex mobility assay (HMA) [38] from 2‐day‐old F0‐generation embryos. The Translate tool (Expasy; SIB, Swiss Institute of Bioinformatics) [39] was used to predict the protein‐level consequences of the mutations. To create an *avd* mutant zebrafish line, F0‐generation embryos were outcrossed with WT Tüpfel long fin (TL) fish and the resulting F1 offspring were screened for desired mutations by sequencing the target site. Indel *avd* ^tpu12^ mutation carrying F1 fish were chosen for usage and further generations of the mutant fish line were created by incrosses. To avoid inbreeding depression, the F5 generation fish were outcrossed to AB WT. Generations F4‐F9 (not including the F6 offspring from the outcrosses) were used in the experiments. Genetic information of the *avd* ^tpu12^ zebrafish line has been submitted to the Zebrafish Information Network (ZFIN) and can be found under an identification code ZDB‐ALT‐241205‐11.

### Zebrafish genotyping

PCR was performed directly from lysed embryos or tailfin tissue using a previously described protocol [40]. The following primers were used for target site amplification Forward: 5′CTCTAATTACTCATTTCCAACAAGG′3 and Reverse: 5′CGCAATATGAAGTTTCAGTAACAC′3. The target site from the *avd* ^tpu12^ zebrafish line was sequenced using an in‐house Sanger sequencing service (Tampere Genomics Facility, Tampere University).

### Liquid chromatography‐coupled mass spectrometry (LC–MS)

Pools of 0‐day‐old embryos (10 embryos/pool, *n* = 4 for both genotypes) from homozygous *avd* ^tpu12/tpu12^ mutant and WT parents were collected and the samples were processed with SPEED method [41]. Briefly, samples were lysed in 100% trifluoroacetic acid reduced with 10 mm Tris‐2‐carboxyethyl‐phosphine and alkylated with 40 mm chloroacetamide. The samples were then neutralized with 2 m Tris base, diluted with water, and digested overnight with sequencing‐grade Trypsin/Lys‐C mix (Promega, Fitchburg, WI, USA). Following digestion, peptides were desalted using a Sep‐Pak tC18 96‐well plate (Waters) and evaporated to dryness.

For LC–MS/MS analysis, samples were reconstituted in 0.1% formic acid, and 800 ng of each sample analyzed on a nanoflow high‐performance liquid chromatography (HPLC) system (Easy‐nLC1200; Thermo Fisher Scientific) coupled to the Orbitrap Exploris 480 mass spectrometer (Thermo Fisher Scientific, Bremen, Germany) equipped with a nano‐electrospray ionization source and FAIMS Pro Duo interface. Peptides were first loaded on a trapping column and subsequently separated on a 15 cm C18 column (75 μm × 15 cm, ReproSil‐Pur 3 μm 120 Å C18‐AQ, Dr. Maisch HPLC GmbH, Ammerbuch‐Entringen, Germany). The mobile phase consisted of water with 0.1% formic acid (solvent A) or acetonitrile/water (80 : 20 (v/v)) with 0.1% formic acid (solvent B). A 120 min gradient was used to elute peptides (62 min from 5% to 21% solvent B followed by 48 min from 21% to 36% solvent B and in 5 min from 36% to 100% of solvent B, followed by 5 min wash stage with solvent B). Samples were analyzed with two FAIMS compensation voltages (CV = −40 and −60 V) using a data independent acquisition (DIA) LC–MS/MS method with one full scan (400–1000 m/z) and 30 DIA MS/MS scans with variable width isolation windows. Data were acquired automatically using the thermo xcalibur 4.7 software (Thermo Fisher Scientific).

Data were analyzed with the spectronaut software (Biognosys, Schlieren, Switzerland; version 21.0) using DirectDIA approach identification and MaxLFQ for label‐free quantification. Main data analysis parameters in spectronaut: Enzyme: Trypsin/P, Missed cleavages: 2, Fixed modifications: Carbamidomethyl, Variable modifications: Acetyl (protein N‐term) and oxidation (M), Protein database: UniProt Danio rerio, Danio rerio Zebavidin mutants 2026_01 and Universal Contaminant Protein database [42], Precursor FDR Cutoff: 0.01, Protein FDR Cutoff: 0.01, MS level Quantification: MS2, Quantification type: Area under the curve within integration boundaries for each targeted ion, Normalization: Local normalization (Based on RT dependent local regression model) described by Callister *et al*., [43].

### Quantitative real‐time PCR

RNA was isolated from pooled samples of 0 dpf (10 embryos/pool) and 5 dpf KO and WT *avd* ^*tpu12*^ embryos (five embryos/pool) using a Nucleospin RNA Plus kit (Macherey‐Nagel, North Rhine‐Westphalia, Germany) and the manufacturer's instructions. For cDNA synthesis, 40–200 ng of RNA from the 0 dpf embryos and 500 ng of RNA from the 5 dpf embryos was taken. cDNA synthesis was done using the High Capacity cDNA Reverse Transcription Kit (Applied Biosystems, Massachusetts, USA). 1/5 diluted cDNA and PowerUp SYBR Green Master Mix (Thermo Fisher Scientific, Massachusetts, USA) were used in the qRT‐PCR reaction, and the analysis was performed using a CFX96™ detection system (Bio‐Rad Laboratories, California, USA). The cfx manager software (v. 3.1; Bio‐Rad Laboratories) was used for the data analysis. Previously described primers were used for the qRT‐PCR; Forward: 5′CGAATGCAAAGGTGAGCTCC′3 and Reverse: 5′ATAGCACGGAGAAAGAGACG′3 [16]. Gene expression was normalized to the *eukaryotic translation elongation factor 1 alpha 1, like 1* (*eef1a1l1* or *ef1a*) [44] expression using the $2^{-\Delta C_{t}}$ method.

### Developmental analyses

Homozygous *avd* ^tpu12^ mutation carrying zebrafish or their WT siblings were bred separately and the eggs collected synchronously at approximately 1 h intervals. The fertilization frequency was estimated by visual inspection of dividing cells under a microscope prior to 6 h post fertilization (hpf). To evaluate the hatching rate, embryos that were alive at 1 dpf were separated and the number of embryos that had hatched at 2, 3, 4, and 5 dpf was recorded. In case an embryo died during the follow‐up, it was removed from the hatching rate analysis. The developmental survival of all fertilized embryos was evaluated and the mortality recorded until 5 dpf.

### Zebrafish imaging

While 1 dpf embryos were imaged without anesthesia, the imaging of the 2–5 dpf embryos and the adult fish was done under 0.02% 3‐amino benzoic acid ethyl ester (Sigma‐Aldrich, St. Louis, MO, USA) anesthetics. Fish were submerged in water during image acquisition. Micrographs from the 1 to 5 days old embryos were taken using an AZ100 Fluorescence Macroscope (Nikon, Tokyo, Japan) and a DFK 33UX250 CMOS color camera (The Imaging Source, Bremen, Germany), whereas adult fish (10‐month‐old) were imaged with an EOS 7D Mark II camera (Canon, Tokyo, Japan).

### Histology

Three‐day‐old KO and WT *avd* ^tpu12^ embryos were euthanized using 0.04% 3‐amino benzoic acid ethyl ester (Sigma‐Aldrich) and fixed in phosphate buffered 10% formalin, pH 7.2 (Reagena, Toivala, Finland) for 1 h at room temperature. The embryos were dehydrated using a gradient of 70% to 100% ethanol with Tissue‐Tek VIP® 6 AI tissue processor (Sakura Finetek, Nagano, Japan) and embedded in paraffin using Tissue‐Tek TEC (Sakura Finetek). Transverse 5 μm sections of the embryos (*n* = 5 for both genotypes) were cut from head to tail using a microtome (Leica, Wetzlar, Germany); the slices were attached onto glass slides using standard procedures and stained using hematoxylin and eosin (H&E) with an Autostainer XL (Leica). The stained sections were imaged using a Nanozoomer S60 digital slide scanner (Hamamatsu Photonics, Shizuoka, Japan) and visualized using an ndp.view 2 software (Hamamatsu Photonics).

### Experimental infections

*S. agalactiae* (FIM314 Serotype III, ST‐17) was cultured as described previously [45]. PBS with 0.5 mg·mL^−1^ phenol red (Sigma‐Aldrich) was used as the carrier solution and 2 nL of the bacterial solution was injected into the embryos. Bacterial inoculations were delivered into the circulation valley of 2 dpf embryos using a PV830 Pneumatic PicoPump (World Precision Instruments, Florida, USA). To confirm the presence of microbes in the inoculates, one injection dose was plated on 5% lamb blood agar plates (Tammer Biolab, Tampere, Finland) before and after the injections and cultivated overnight at 37 °C with 5% CO_2_.

### Statistical analyses

graphpad prism (v. 10.3.1, Dotmatics, Boston, Massachusetts) was used for the statistical analyses. A two‐tailed Mann–Whitney test, unpaired Student's *t*‐test with combined MS1 + MS2 statistical model [46] and multiple‐testing correction of the *P*‐values with Benjamini–Hochberg method and a log‐rank (Mantel‐Cox) test were used for the statistical comparison of differences. *P* < 0.05 was considered statistically significant.

## Results

### Avidin‐like proteins exist in both bony and cartilaginous fish

Although the presence of several avidins and avidin‐related proteins has been reported in egg‐laying vertebrates, such as birds and amphibians [3, 10, 11], the current knowledge on fish avidins is scarce [15, 16]. To identify avidin‐encoding genes and avidin‐like proteins in fish species, we used verified Uniprot‐derived amino acid sequences of zebrafish (E7F650), chicken (*Gallus gallus*, P02701), *X. tropicalis* (A7YYL1) and *S. avidinii* (P22629) avidins and screened for orthologous genes and homologous proteins in jawless (Agnatha; 8 genomes), bony (Actinopterygii; 1135 genomes, Sarcopterygii; 3 genomes), and cartilaginous fish (Chondrichthyes; 25 genomes) using BLASTp and tBLASTn [35]. While our search did not find *avd* genes or avidin‐like proteins in jawless fish, we obtained 550 hits with BLASTp (E7F650: 164 hits, P02701: 163 hits, A7YYL1: 161 hits, and P22629: 62 hits), and 568 hits with tBLASTn (E7F650: 179 hits, P02701: 174 hits, A7YYL1: 165 hits, and P22629: 50 hits) that belonged to the other fish classes. After the removal of overlapping hits based on their IDs, we ended up with 164 hits with BLASTp and 192 hits with tBLASTn (Table S1). The final list of 356 hits was curated to include 130 avidins from 92 fish species once duplicate proteins and highly similar isoforms were omitted (Table S2). More specifically, single copy of avidin genes/proteins was identified in 63 species (e.g., *Danio rerio*), two homologs in 20 species (e.g., *Labeo rohita*) and three or more avidins in 9 species (e.g., *Latimeria chalumnae*).

**Fig. 1:**
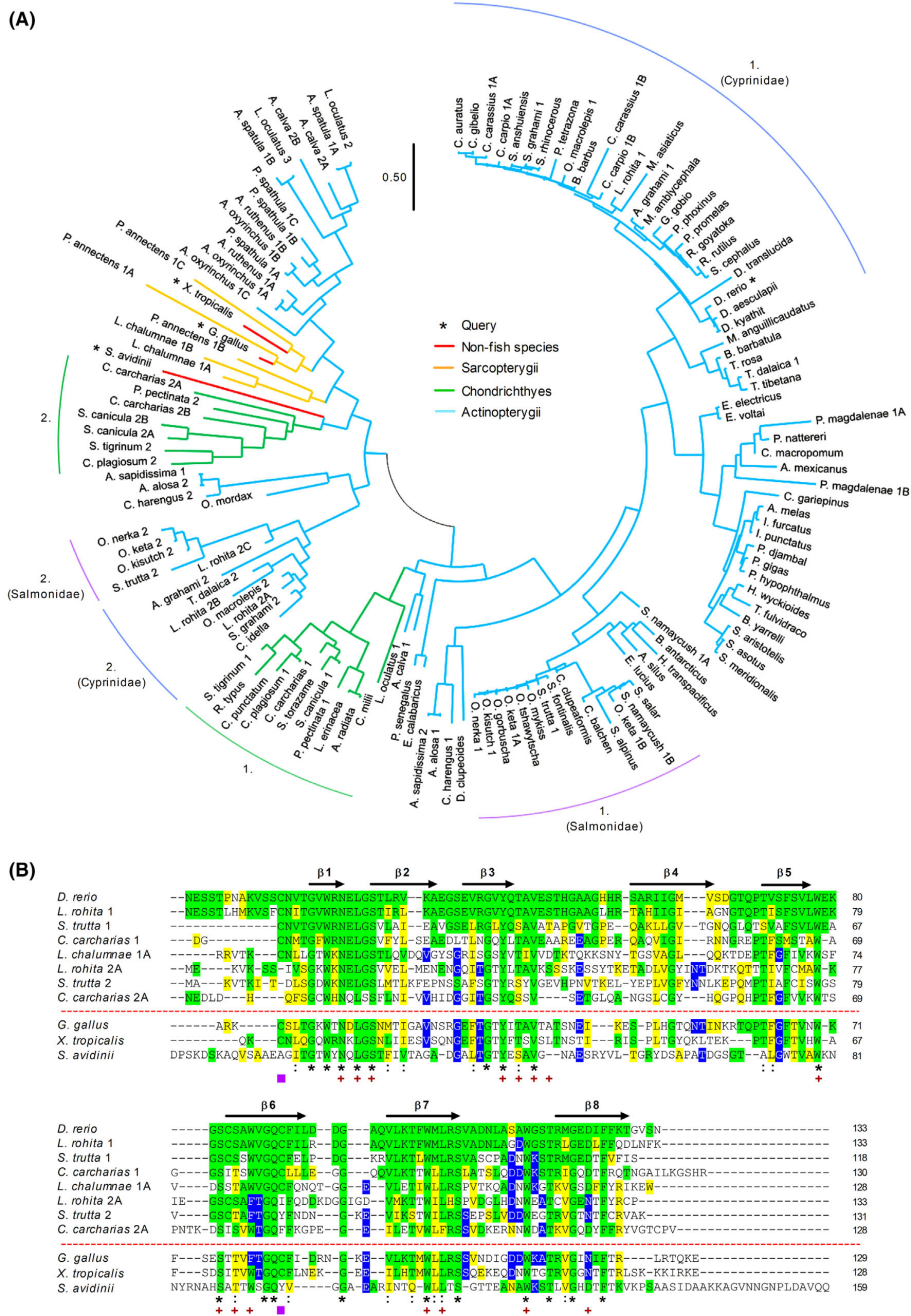
Avidin‐like proteins are evolutionarily conserved in fish species. Zebrafish (E7F650), chicken (*Gallus gallus*, P02701), *X. tropicalis* (A7YYL1) and *S. avidinii* (P22629) avidin amino acid sequences were used as references, and 130 unique putative avidins were identified in bony (Actinopterygii and Sarcopterygii) and cartilaginous fish (Chondrichthyes) using BLASTp and tBLASTn. (A) The query avidins and the identified fish proteins were used to construct a phylogenetic tree using Molecular Evolutionary Genetics Analysis (MEGA). The two clades of avidins within Chondrichthyes (class) as well as Salmonidae (family) and Cyprinidae (family) are numbered in the circle periphery. (B) Seven novel avidins were selected from the major fish classes (Actinopterygii, Sarcopterygii and Chondrichthyes) and aligned with the query avidins using MEGA. Green depicts identical and yellow similar amino acids to zebavidin, whereas blue shows similarity to chicken avidin. Above the alignment, the typical beta‐sheet (β1‐8) forming amino acids of avidin are depicted using arrows. Asterix = fully conserved residues, Colon = highly conserved residues, Purple square = disulfide bridge forming Cys13 and Cys90, Red plus sign = biotin‐binding residues.

Next, we performed a multiple sequence alignment of the identified avidins (or translated *avd* genes) and studied their evolutionary relationships by constructing a phylogenetic tree. Here, we expectedly saw that zebavidin (labeled as *D. rerio*) clustered together with avidins identified in other species of the Cyprinidae family (class: Actinopterygii—ray‐finned fish), whereas the chicken, *X. tropicalis* and *S. avidinii* avidin proteins clustered with Sarcopterygii (lobe‐finned fish) and Chondrichthyes (cartilaginous fish) class avidins (Fig. 1A). Two clades of avidins were identified within Chondrichthyes as well as within the Cyprinidae and Salmonidae families of Actinopterygii, demonstrating that, unlike zebrafish, certain fish species have multiple avidin/avidin‐like analogs (e.g., avidins 1, 2A and 2B from *Carcharodon carcharias*—the great white shark). In fact, the alignment of selected avidins from *D. rerio* (Cyprinidae, Actinopterygii), *L. rohita* (Cyprinidiae, Actinopterygii), *S. trutta* (Salmonidae, Actinopterygii), *C. carcharias* (Lamnidae, Chondrichthyes), and *L. chalumnae* (Latimeriidae, Sarcopterygii) revealed that while the amino acid residues that directly interact with biotin in chicken avidin (such as Tyr40, numbering done according to the primary structure of zebavidin) are identical or well‐conserved, certain features, such as the disulfide bridge‐forming residues Cys13 and Cys90, that are present in zebavidin, chicken avidin and in the avidins of the first clade, are missing in the homologs of the second clades, namely *L. rohita* 2A, *S. trutta* 2 and *C. carcharias* 2A [16, 47] (Fig. 1B). Our analysis also indicated that the second clade avidins and *L. chalumnae* avidin 1A shared more residues with the non‐fish homologs than the clade 1 avidins.

More detailed analysis of the multiple sequence alignment of fish avidins revealed several highly conserved glycine residues that, to our knowledge, have not been studied previously. The most conserved glycines were Gly17, Gly24, and Gly38, and they may enable tight turns between the β‐strands that form the characteristic avidin β‐barrel [16, 48]. High conservation was also observed for residues Tyr40, Trp78, Trp86, Trp104, and Trp116 that are known to be important for high biotin‐binding affinity in chicken avidin [47] and bacterial streptavidin [49] and have been shown to contribute to biotin binding from an adjacent subunit [50, 51], thus suggesting similar oligomeric (tetrameric) assembly for the majority of fish avidins. We noticed that certain residues in putative fish avidins are exclusively hydrophobic: Val15, Leu27, Val29, Val36, Val44, Ile58, Val71, Phe73, Val75, Phe91, Leu100, Leu105, Leu106, and Phe126, which may also affect protein folding and assembly of oligomers.

Collectively, our data indicate that fish avidins are widely expressed in fish species, share functionally important amino acid residues involved in high‐affinity biotin binding, characteristic beta barrel structure with tight turns, and oligomeric assembly. Putative fish avidins thus display substantial similarity to their non‐fish homologs in structurally and functionally important residues across the sequence. Additionally, our data suggest that, similarly to the chicken, where the avidin gene family appears to contain several members potentially encoding functional proteins [52, 53, 54], gene families with multiple avidin analogs also exist in fish species.

### CRISPR/Cas9 mutagenesis of the zebrafish *avd* gene

**Fig. 2:**
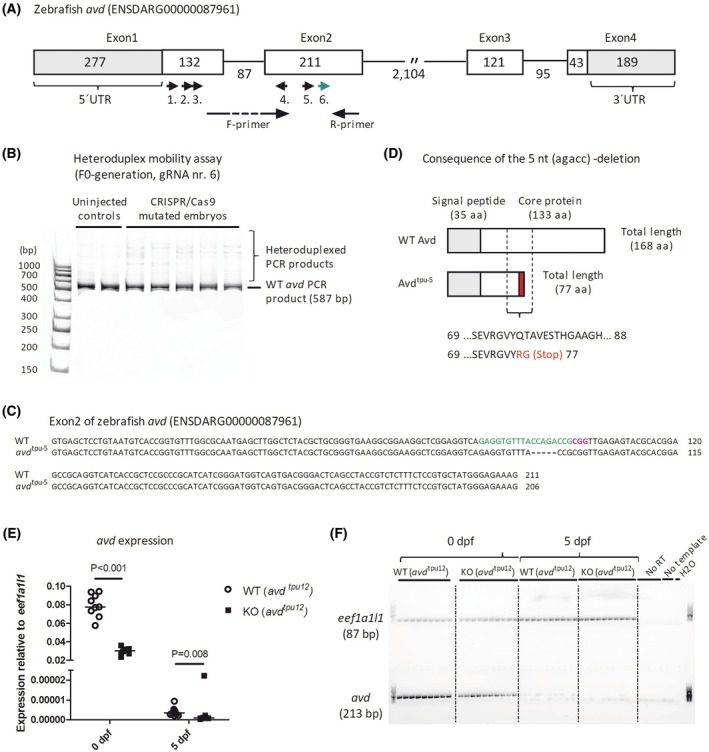
*Avd* ^tpu12^ zebrafish harboring a nonsense *avd*‐mutation was created using CRISPR/Cas9 mutagenesis. (A) A schematic representation of the zebrafish *avd* gene (ENSDARG00000087961). Exonic regions are depicted as boxes, whereas introns are shown as connecting lines. Arrows indicate the orientation and the positioning of the guide RNAs (gRNAs) and the qPCR primers used in the study. A green arrow indicates the functional gRNA used to create *avd* KO zebrafish. (B) A heteroduplex mobility assay was used to estimate the efficiency of mutagenesis at the *avd* target site in 2‐day‐old embryos. While a single wild‐type (WT) *avd* PCR product is seen in the uninjected controls, heteroduplexed PCR products can be detected in the gRNA 6. and Cas9 co‐injected embryos, indicating successful mutagenesis. (C) The DNA sequence of the second exon of the *avd* gene (ENSDARG00000087961) is represented schematically in WT and in *avd* ^tpu12^ mutant zebrafish. The gRNA target site (nr. 6) is depicted in green letters and the protospacer adjacent motif (PAM) in purple. (D) Schematics of the 168 amino acid (aa) wild‐type (WT) protein and the 77 aa Avd^tpu12^‐mutation (deletion of agacc) ‐harboring form are depicted. The premature stop codon on the primary aa sequence of the mutated zebavidin is also shown. (E) qRT‐PCR was used to quantify the relative expression of *avd* mRNA in *avd* knockout (KO) and in WT embryos at 0 and 5 days post fertilization (dpf). (F) The specificity of the qRT‐PCR products was analyzed using agarose gel electrophoresis (1.5% w/v agarose gel in water). No reverse‐transcriptase (no RT), no template controls (no template in reverse transcription) and H_2_O controls (no template in the PCR reaction) were used to exclude DNA/PCR contaminations. A two‐tailed Mann–Whitney test was used in the statistical comparison of differences in panel E.

With the advent of the CRISPR/Cas9 technology, we have previously knocked out genes of interest in zebrafish to understand their gene‐function relationships *in vivo* [31, 32, 33]. To knock out zebrafish *avd* (ENSDARG00000087961), we designed and produced six gRNAs that targeted the translated regions of both the first and the second exons of the gene (Fig. 2A) and studied their ability to induce mutations in zebrafish embryos following a co‐injection with the Cas9 protein. While a heteroduplex mobility assay revealed that five out of the six gRNAs did not induce gross mutagenesis at the corresponding target sites, gRNA number 6 showed apparent heteroduplex formation on PAGE, indicating a detectable frequency of targeted indel mutations upon injection of this gRNA (Fig. 2B). Crossing adult F0‐generation mutants with WT zebrafish and genotyping their F1‐offspring demonstrated that *avd* mutations are also transmitted to the germline cells. Importantly, in the F1‐generation fish, we identified a 5‐nucleotide deletion (agacc) that alters the reading frame of the *avd* gene and creates a truncated 77 amino acid avidin protein (Avd^tpu12^) with 75/168 amino acids shared with the WT protein (Q76 and T77 modified to R76 and G77 at the C terminus of the mutant protein) (Fig. 2C,D). Such a deletion destroys the ability of zebavidin to bind biotin with reasonable affinity and is also likely to block the formation of oligomers that are important for high‐affinity biotin binding. The loss‐of‐function *avd* ^tpu12^ mutation was further confirmed using LC–MS. Here, our analysis identified six different zebavidin peptides from WT embryos, including one that spans the CRISPR/Cas9 cut site, whereas only two peptides were found in the *avd* ^tpu12/tpu12^ mutants (Fig. S1, Table S3). Also, in line with our previously characterized *itln3* and *pcsk9* KO zebrafish lines [31, 32], we saw decreased *avd* expression in homozygous *avd* ^tpu12^ mutants in comparison with the WT fish at 0 and 5 dpf, suggesting nonsense mediated mRNA decay in the KO embryos (Fig. 2E,F).

### Zebavidin is dispensable for zebrafish development

**Fig. 3:**
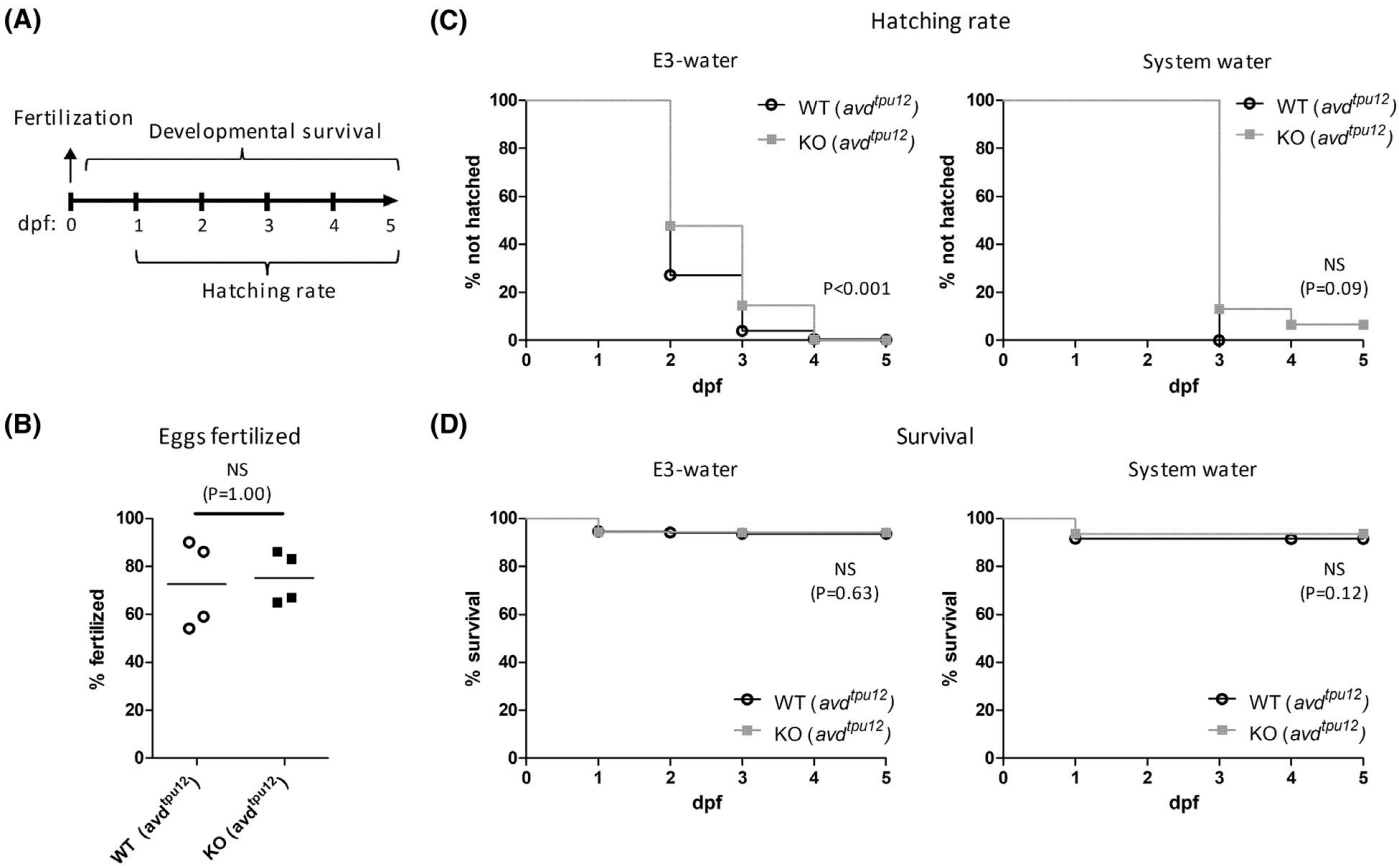
Fertilization, hatching rate and survival of *avd* knockout (KO) zebrafish is comparable to wild‐type (WT) controls. (A) A schematic illustration of the timeframe of the developmental analyses of the study. (B) The frequency of fertilized eggs in *avd* KO and WT embryos was recorded in four independent experiments and the percentage is depicted as a scatter dot plot with median. (C) Embryos were grown in E3‐ or system water and the frequency of hatched embryos was evaluated in *avd* KO (E3‐water: *n* = 549, system water: *n* = 23) and WT embryos (E3‐water: *n* = 918, system water: *n* = 21) between 1 and 5 dpf. Data are representative of three independent experiments (see also Fig. S2). (D) Embryos were grown in E3‐ or system water and the survival during development was recorded in developing *avd* KO (E3‐water: *n* = 582, system water: *n* = 653) and WT embryos (E3‐water: *n* = 976, system water: *n* = 933) by removing non‐fertilized eggs and recording the mortality daily prior 5 dpf. Data are representative of three independent experiments (see also Fig. S2). In panel B, a two‐tailed Mann–Whitney test was used in the statistical comparison of difference, whereas a log‐rank (Mantel‐Cox) test was used in panels C and D.

An avidin‐rich diet, leading to biotin deficiency, has been shown to impair the growth and survival of different vertebrates, including fish species, such as rainbow trout (*Oncorhynchus mykiss*), Nile tilapia (*Oreochromis niloticus*), and zebrafish [6, 7, 8], which reflects the important role of biotin as a cofactor in lipid metabolism. However, there are no studies exploring the effects of *avidin* KO in any species. To study the effects of the absence of zebavidin on the developing zebrafish embryos, we first recorded the fertilization rate, hatching rate, and survival of the *avd* KO and WT embryos (Fig. 3A). Moreover, hatching and survival were monitored both in E3‐water and water from our zebrafish facility flow‐through system to get insights into the developmental role of zebavidin in microbiologically different environments. In our experimental setup, instead of using heterozygous *avd* ^tpu12/+^ fish as parents, we incrossed homozygous *avd* ^tpu12/tpu12^ fish to obtain KO embryos and WT *avd* ^tpu12^ fish to obtain WT embryos for the experiments. This ensured the absence of maternal zebavidin or *avd* mRNA in KO embryos. While we observed comparable fertilization rates between the *avd* KO and WT eggs with median frequencies of 75% and 73%, respectively (Fig. 3B), the absence of zebavidin was associated with a modestly decreased hatching rate in E3‐water (*P* < 0.001) (Fig. 3C and Fig. S2A). Such a difference was, however, not seen in eggs kept in system water (Fig. 3C and Fig. S2B). When the survival of *avd* KO and WT embryos in these two conditions was monitored for 5 days, no significant differences were seen between the lines in E3‐water (94% survival of *avd* KO fish and 94% of WT fish) or in system water (94% survival of *avd* KO fish and 91% of WT fish) (Fig. 3D and Fig. S2C,D). Overall, we conclude that the lack of zebavidin does not affect the fertilization, hatching, or survival of zebrafish embryos.

**Fig. 4:**
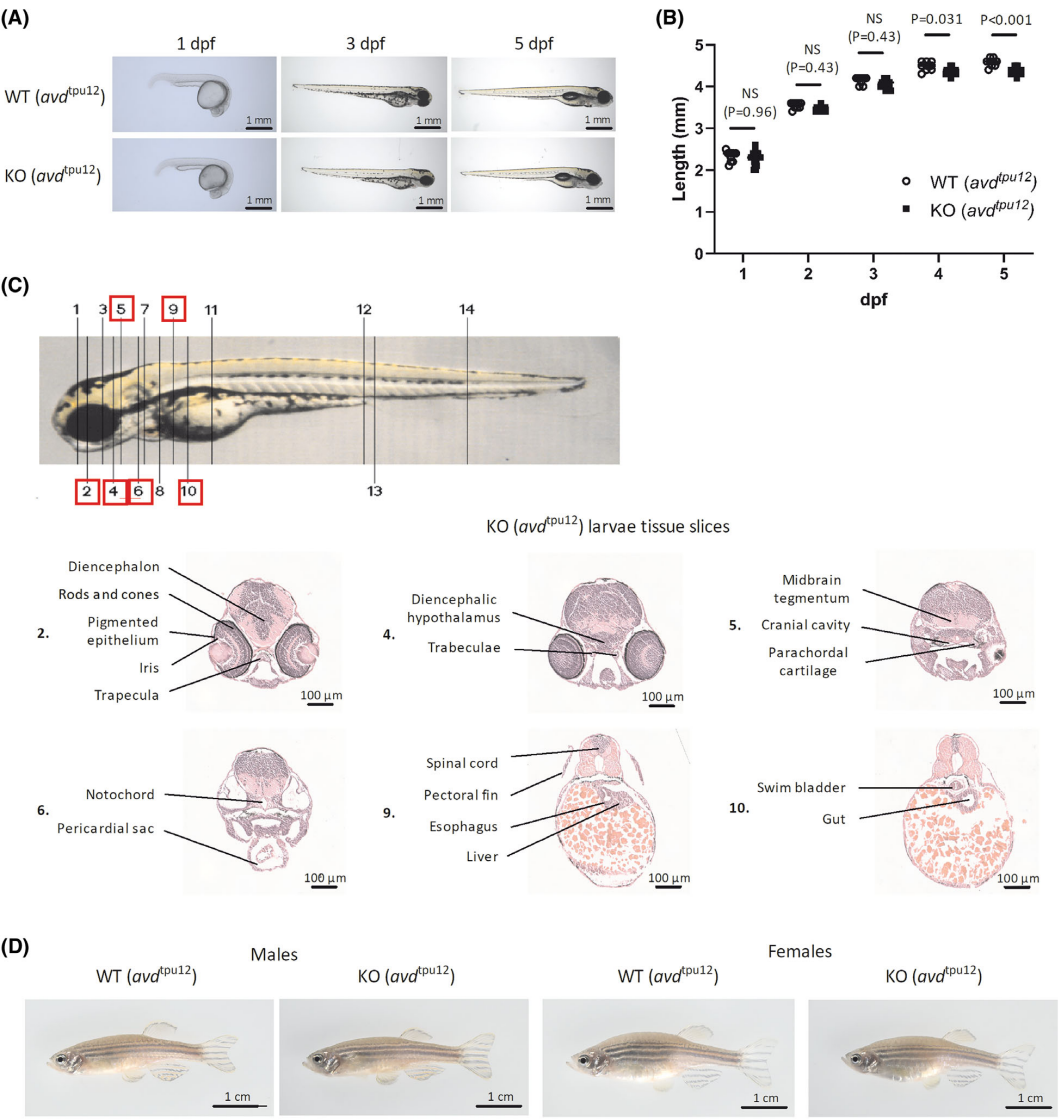
Morphology of *avd* knockout (KO) zebrafish embryos and adult fish is similar to wild‐type (WT) controls. (A) Embryos were grown in E3‐ or system water and *avd* KO (*n* = 10 in both conditions) and WT embryos (*n* = 5 in both conditions) were imaged at 1, 3, and 5 days post fertilization (dpf) using an AZ100 Fluorescence Macroscope (Nikon). Representative embryos grown in E3‐water are shown. (B) Length of the embryos between 1 and 5 dpf was measured from head to tail using inkscape v. 1.4.2. Both E3‐ and system water grown embryos are shown in the plot. (C) *avd* KO (*n* = 5) embryos were prepared for histology at 3 dpf. Horizontal view of a 3‐day‐old embryo micrograph from the Zebrafish histology atlas (https://zfin.org/zf_info/anatomy/72hrs/72hrs.html) is shown on the top as a reference and longitudinally cut HE‐stained tissue slices from representative *avd* KO embryos are shown below. Recognizable anatomical structures within specified regions (red boxes, numbers 2, 4, 5, 6, 9. and 10) have been named. (D) Ten‐month‐old adult zebrafish (*n* = 4; 1 female, 3 males in WT and *n* = 5; 2 females, 3 males in *avd* KO fish) were imaged with EOS 7D Mark II camera (Canon), and representative fish from both genotypes are shown. In panel B, Kruskal–Wallis test was used for the statistical comparison of differences. During image acquisition, fish were submerged in water and anesthetized from 3 dpf onwards.

In mice, it has been demonstrated that an avidin‐induced biotin‐deficiency can lead to malformations during embryonic development [5]. Consequently, we next studied the morphology of the *avd* KO embryos and the adult fish using imaging. Overall, *avd* KO embryos showed no signs of malformation or delay in development during the first 5 dpf (Fig. 4A), although the KO embryos were shorter than the WT controls at 4 dpf (4.4 mm vs. 4.5 mm, respectively, *P* = 0.031) and 5 dpf (4.4 mm vs. 4.6 mm, respectively, *P* < 0.001) (Fig. 4B). Histological analysis of 3 dpf embryos revealed no gross anatomical differences in the nervous system, digestive tract, eye, liver, heart, swim bladder nor in structural components compared with the reference embryos (Fig. 4C), further indicating that both the temporality and the spatiality of development are intact in the absence of zebavidin. Also, in the adult zebrafish, no anatomical differences were observed between KO *avd* ^tpu12^ and WT fish at the age of 10.5 months (Fig. 4D). In addition, both KO and WT fish showed comparable behavior and breeding ability during their maintenance indicating that zebavidin is dispensable also later in life. Together with the results obtained from fertilization, hatching and early survival studies, these morphological analyses suggest that zebavidin is dispensable for zebrafish development and growth under laboratory conditions.

### Resistance against *S. agalactiae* is not altered in *avd* KO embryos

**Fig. 5:**
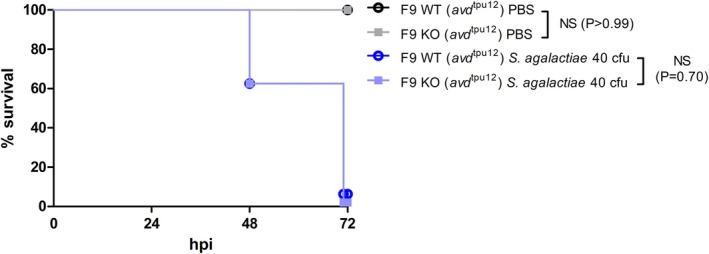
Zebavidin is dispensable in *S. agalactiae* infection of zebrafish embryos. F9‐generation *avd* knockout (KO) and wild‐type (WT) embryos were microinjected into the circulation valley with saline solution (PBS, *n* = 12 for both genotypes) or with 40 cfu of *S. agalactiae* (*n* = 48 for both genotypes) at 2 days post fertilization (dpf) and the survival of the embryos was recorded until 5 dpf. A log‐rank (Mantel‐Cox) test was used for the statistical comparison of differences.

While the previously reported avidin‐induced developmental defects are likely caused by reduced levels of biotin in the organisms, the ability of avidin to decrease the bioavailability of biotin from its surroundings has been proposed to be beneficial in microbial defense [20]. In fact, the differential expression of genes regulating biotin biosynthesis has been associated with the pathogenicity of *S. agalactiae* in tilapia [55]. Since *S. agalactiae* is a natural fish pathogen and we and others have previously shown that also zebrafish are susceptible to *S. agalactiae* infection [45, 56], we used an experimental *S. agalactiae* infection in zebrafish to study the effect of zebavidin on the host defense against bacterial exposure. 40 colony forming units (cfu) of *S. agalactiae* were microinjected into the blood stream of 2 dpf *avd* ^tpu12^ KO and WT embryos and their survival was monitored for 3 days. We did not see any significant differences in the survival curves or endpoint survival percentages between the *avd* KO and WT embryos (2% KO embryos vs. 6% WT embryos) (Fig. 5). This result indicates that the resistance against systemic *S. agalactiae* infection in zebrafish is unaltered in the absence of zebavidin.

## Discussion and conclusions

Observations on the toxicity of a raw egg white diet led to the identification of a biotin‐binding protein, named avidin (derived from ‘avidity for biotin’) already in the early 1900s [2]. Since then, the high‐affinity, non‐covalent avidin‐biotin interaction (*K*_D_ ~ 10^−15^ moL·L^−1^) has been adapted to numerous biotechnological applications, such as protein purification and detection [57]. To date avidins or avidin‐like proteins have been found in bacteria [14], fungi [12] as well as in several egg‐laying vertebrates, such as chicken, frogs and turtles [2, 3, 10]. While an avidin homolog was previously identified also in zebrafish [15], to our knowledge, the presence of *avd* genes in other fish species or their phylogenic relationships have not been comprehensively studied. In the current study, we used amino acid sequences of avidins from zebrafish, chicken, *X. tropicalis* and *S. avidinii* and used BLAST to search for avidin‐like genes and homologous proteins in fish species. Our data, consisting of 130 putative avidins, demonstrates that avidins are evolutionarily conserved in lobe‐finned fish (Sarcopterygii), cartilaginous fish (Chondrichthyes) and ray‐finned fish (Actinopterygii) and display high similarity in amino acids involved in biotin‐binding and several other residues characteristic for avidin structure in comparison with each other and to query avidins. Moreover, while a single avidin protein has been identified in zebrafish, more than two avidin homologs were identified in 29 fish species, including *L. rohita* and *C. carcharias*, which suggests similarity to, for example, chickens that can produce both prototype avidin and avidin‐related proteins [52, 53, 54]. Further studies are required to determine the biochemical properties and the biological significance of the fish avidins. For example, calculating the ratio of nonsynonymous substitutions per nonsynonymous site (dN) to synonymous substitutions per synonymous site (dS) could be used to elucidate whether the *avd* genes in fish species are under diversifying (positive) or purifying (negative) selection.

The morpholino knockdown technology has been widely used in zebrafish embryos to silence genes of interest. In fact, we have used this system to demonstrate that knocking down *avd* using translation‐blocking morpholinos did not affect embryonic development prior to 48 hpf [16]. There are, however, certain previously reviewed limitations that should be acknowledged when using morpholinos [58, 59]. Firstly, off‐targeting of morpholinos can lead to the activation of p53‐mediated cell death pathways and subsequent developmental defects [60]. Secondly, the efficiency of the knockdown gradually decreases during development, which eventually leads to restoration of the target gene's function [27]. Lastly, while the splice‐blocking morpholinos can cause predictable mRNA alterations, that can be detected using, for example, PCR, evaluating the knockdown efficiency of translation‐blocking morpholinos is difficult in zebrafish due to the scarcity of commercial antibodies [58]. By creating *avd* KO zebrafish, we were able to study the role of zebavidin throughout the development of the embryos until adulthood. We were able to breed the *avd* KO zebrafish (homozygous for *avd* ^tpu12^) and use their offspring to study whether maternally deposited *avd* mRNA (or the zebavidin protein) in the eggs affected fertilization or the early stages of development that occur prior the maternal to zygotic transition at 6 hpf [61]. In our work, we provide genetic, mRNA and protein‐level information on the effects of the *avd* ^tpu12^ mutation. In line with expectations, we observed substantial decrease in *avd* mRNA expression. Additionally, MS analysis identified avidin peptides across the protein sequence from the WT samples, but only signs of avidin fragments, likely arising from exon skipping or alternative translation initiation codon, were observed in eggs collected from the *avd* KO fish. Such polypeptides would only form partial β‐barrel, leaving significant hydrophobic patches exposed, thus likely representing poorly soluble, aggregated isoform that accumulates into the cytoplasm. Also, although other avidin‐like genes have not been identified in zebrafish, the possible compensatory gene expression pathways, that have been reported in other *in vivo* gene KO studies [62], should later be studied in *avd* KO zebrafish.

Biotin deficiency in humans can cause clinical manifestations that include rash, alopecia and developmental delay [63]. Previous studies in fish species have demonstrated that an excess of dietary avidin causes a pathological biotin‐deficient condition characterized by growth retardation, anemia and increased mortality [6, 7, 8]. Furthermore, supplementation of maternal avidin has been shown to exhibit a teratogenic effect on fetuses in mice, suggesting that biotin availability is crucial also for embryonic development [5]. But whether the absence of avidin affects embryonic development has not been previously addressed. Here, we conducted several functional (fertilization rate, hatching rate, and survival) and morphological (live imaging and histology) analyses and show that knocking out *avd* does not impact zebrafish development. Consequently, it can be hypothesized that zebavidin is well‐tolerated at physiological concentrations but exceeding a given threshold is harmful. It is also possible that we have missed a physiological phenotype that is not seen as gross changes in morphology or that the well‐controlled laboratory environment is not sufficient to unravel the phenotype caused by the absence of zebavidin. In fact, in the laboratory zebrafish are kept at a relatively stable pH, temperature and salinity and the water is constantly exchanged or filtered and treated with ultraviolet light, which can collectively have a major impact on the importance of gene/protein of interest during development. The localization of the protein can also impact its functionality in the embryos, as it has been recently reported that zebavidin is most abundant in the egg yolk but can also be found in the perivitelline fluid and in the chorion [64]. In chicken eggs, availability of biotin to developing embryo is enabled by yolk biotin‐binding proteins resembling avidin [65, 66], whereas avidin with extremely high biotin‐binding affinity is located in the egg white [67, 68]. Therefore, it is possible that the moderate affinity of zebavidin–biotin interaction (*K*_D_ ~3 × 10^−9^ m) enables biotin supplementation to developing embryos in specific conditions. It is also noteworthy that large batch‐to‐batch variation in the hatching rate of the embryos was observed in our experiments, indicating that undefined parental or environmental contribution [69] can play a major role in developing zebrafish and should be well‐controlled in experimental settings.

Both sterile inflammation and an *Escherichia coli* infection have been shown to induce avidin expression *in vivo* in the chicken oviduct and intestine [17, 23]. This, together with the ability of avidin to sequester biotin from its surroundings, suggests that avidin is a host response protein against pathogens or competing micro‐organisms. We and others have shown that zebrafish are susceptible to *S. agalactiae* infection as indicated by the ability of bacteria to multiply within the host and translocate into the brain upon an experimental vascular injection [45, 56]. Additionally, a relatively recent paper described that the up‐regulation of biotin synthase (BioB) in an *S. agalactiae* strain, NNA048, was associated with higher pathogenicity in tilapia [55] thereby bridging the bioavailability of biotin with the severity of an *S. agalactiae* infection. To evaluate whether *avd* KO in zebrafish embryos affects the virulence of *S. agalactiae* or the host response, we injected the bacteria into the circulation valley of zebrafish embryos at 48 hpf. The comparable survival between *avd* KO and WT embryos suggests that the absence of zebavidin does not impair zebrafish resistance against the pathogen. Further research is required to understand whether the ability of certain bacterial species to *de novo* biosynthesize biotin enables the bacteria to overcome the avidin‐mediated effects of biotin‐binding in the WT embryos. Also, keeping in mind that avidin has been reported to have both antifungal and insecticidal properties [70, 71], studying zebavidin in other infection models, with fungi or parasites, could better explain the biological significance of this protein in host defense.

Our study provides novel information on the phylogeny of fish avidins and demonstrates that *avd* genes are found in both bony and cartilaginous fish species. We observed 130 putative avidins, and the phylogenetic analysis and sequence conservation link them structurally with the previously characterized avidins. We used CRISPR/Cas9‐mediated mutagenesis to additionally demonstrate that *avd* in zebrafish is dispensable for fertilization, embryonic development, and fish growth under laboratory conditions. Lastly, our data imply that the absence of zebavidin in zebrafish embryos is not detrimental upon an *S. agalactiae* challenge. Taken together, we provide novel information on the phylogeny and biology of fish avidins, both of which are research areas that are still largely unexplored. Furthermore, the developed *avd* KO fish model could be a useful tool for understanding the importance of avidin as a protective factor against other invaders, such as eukaryotic parasites.

## Conflict of interest

The authors declare no conflict of interest.

## Author contributions

A.K.S., M.J.T.O., M.P., M.R., and V.P.H. conceived and designed the project. A.K.S., M.J.T.O., and O.K. acquired the data. A.K.S., M.J.T.O., O.K., M.P., M.R., and V.P.H. analyzed and interpreted the data. A.K.S., M.J.T.O., O.K., M.P., M.R., and V.P.H. wrote the manuscript.

## Supporting information


**Fig. S1.** Liquid chromatography ‐coupled mass spectrometric (LC–MS) analysis of zebavidin peptides in zebrafish embryos.
**Fig. S2.** Data from individual experiments of hatching rate and developmental survival analyses.

**Table S1.** BLASTp and tBLASTn hits with unique identifiers (ID).

**Table S2.** Curated hits from BLAST analysis.

**Table S3.** Liquid chromatography ‐coupled mass spectrometric (LC–MS) identification of zebavidin peptides in zebrafish embryos.

## Data Availability

Generated and analyzed data are available upon reasonable request from the corresponding author.
