# Supplementary material for: Avidin is evolutionarily conserved in fish but dispensable for development and resistance against Streptococcus agalactiae in zebrafish
Source: FEBS Open Bio. 2026 Jul 31:10.1002/2211-5463.70320. Online ahead of print. doi: 10.1002/2211-5463.70320 (PMC13425791; doi:10.1002/2211-5463.70320)
Supplement: Supplementary file 1 — Fig. S1. Liquid chromatography ‐coupled mass spectrometric (LC–MS) analysis of zebavidin peptides in zebrafish embryos. Fig. S2. Data from individual experiments of hatching rate and developmental survival analyses. [file FEB4-9999-0-s002.docx]

**Supplementary figures**

Avidin is evolutionarily conserved in fish but dispensable for development and resistance against *Streptococcus agalactiae* in zebrafish

**Authors:** Anni K. Saralahti^1,*^, Markus J.T. Ojanen^1,*,#^, Mataleena Parikka^1^, Otto Kauko^2^, Mika Rämet^1^, Vesa P. Hytönen^1,3^

**Affiliations:**

^1^Faculty of Medicine and Health Technology, Tampere University, Tampere FI-33520, Finland;

^2^Turku Bioscience Centre, University of Turku and Åbo Akademi University, Turku FI-20520, Finland

^3^Fimlab Laboratories, Tampere FI-33520, Finland

**Footnotes:**

*Equal contribution

**^#^**Correspondence to Dr. Markus Ojanen, Faculty of Medicine and Health Technology, Tampere University, Arvo Ylpön katu 34, FI-33520 Tampere, Finland, Phone: +358401909728, Fax: +358033641053, Email address: markus.ojanen@tuni.fi

**Supplementary Figure 1. Liquid chromatography -coupled mass spectrometric (LC-MS) analysis of zebavidin peptides in zebrafish embryos.** A) Pools of 0-day-old embryos (10 embryos/pool, n=4 for both genotypes) from homozygous *avd*^tpu12/tpu12^ mutant and wild type (WT) parents were collected and analyzed for the presence of zebavidin peptides using liquid chromatography (LC) -coupled mass spectrometry (MS). Data is represented as a scatter dot-plot with median using a logarithmic (log10) y-axis. Unpaired Student’s t-test with combined MS1 + MS2 statistical model (Huang et al., 2019) and multiple-testing correction of the p-values with Benjamini-Hochberg method was performed for comparisons between WT (*avd*^tpu12^) and knockout (KO) (*avd*^tpu12^) embryos. N/A=P-value could not be calculated due to undetectable levels of the peptides in the KO fish. B) The identified six peptides within WT zebavidin protein are highlighted in green. The signal peptide region has been excluded from the amino acid sequence.

**Supplementary Figure 2. Data from individual experiments of hatching rate and developmental survival analyses. A-B)** Embryos were grown in E3- and system water and the frequency of hatched embryos recorded in *avd* knockout (KO) (E3-water: n=482 (Exp. #1), n=549 (#2) and n=45 (#3), system water: n=700 (#1), n=611 (#2), n=23 (#3)) and in wild type (WT) embryos (E3-water: n=566 (#1), n=918 (#2) and n=40 (#3), system water: n=288 (#1), n=854 (#2), n=21 (#3)) between 2 and 5 dpf. **C-D)** Embryos were grown in E3- and system water and the developmental survival was evaluated in *avd* KO (E3-water: n=495 (Exp. #1), n=582 (#2) and n=46 (#3), system water: n=954 (#1), n=653 (#2) and n=25 (#3)) and in WT embryos (E3-water: n=606 (Exp. #1), n=976 (#2) and n=40 (#3), system water: n=311 (#1), n=933 (#2) and n=28 (#3)). Non-fertilized eggs were removed from the analysis and the mortality recorded daily until 5 dpf. Note that representative images of these experiments are shown in Figure 3. A log-rank (Mantel-Cox) test was used for the statistical comparison of differences.
